# The Hospitals' Future and Medical Practice

**Published:** 1918-10-19

**Authors:** 


### The Hospital

The Workers' Newspaper of Administrative Medicine and Institutional Life, Administration, National Insurance and Health.

No. 1689, Vol. LXV. SATURDAY, OCTOBER 19, 1918. PRICE TWOPENCE

### THE HOSPITALS' FUTURE AND MEDICAL PRACTICE.

Many readings are necessary to an adequate appreciation of the matter that is in Sir George Newman's memorandum on medical education, and each reading gives new thoughts, for every paragraph contains a text on which much can be said in exposition. The memorandum is of greater importance to the medical profession than the profession has yet completely understood. It is an omen of a new era, and, let us hope, a better era. Let it not be imagined that in a year or two there will be upset of the present and a sudden start on a future. Revolutions are matters of years, though each dates from its own Bastille. But it may be anticipated that the momentum imparted by this document will hasten the evolution of the practice of medicine.

A sudden revolution is not possible, whilst the medical profession exists as it now is, the members of it are on old lines, and most of them fall far behind Sir George Newman's high ideals of what the attainments and virtues of a practitioner of medicine should be. Still, the Register cannot be scrapped and replaced immediately. The men now on it are the machinery with which must be done the work that at present entails on the profession and the further work that, according to the signs of the times, will be demanded of it. The various prejudices of the public also will be a drag strong enough to prevent sudden drastic change.

It would seem, too, that before the revolution can be at full blast the new teachers to train the new men must be found and trained. In many of the great medical schools there are some teachers that truly inspire and that approach Sir George's high ideals, but they would form but a little company. Still, Sir George's source of inspiration appears to be the Report issued by the University of London's Commissioners. It is one of the objects of the memorandum to set forth this last statement, and it is doubtful if enough men with the character, capacity, and genius to reach near the ideal can at present be found. Superlative gifts are rarely granted. Sir George's ideal demands them.

There is no intention to make captious criticism, but it does seem that Sir George hopes too much from the power of the teacher to mould the taught. The teacher can and should set the high ideal and expound it, but only those who have the inherited intellectual power and the birth-given spiritual fire can reach the height of great endeavour. Men can be taught up to the capacity they are born with; teachers cannot give capacity. If the training and the test are to be such as can be successfully completed and complied with only by the very able, men of second-class intellect will not face the pecuniary risks of failure. If the demand is to be for the higher order of intellect only, the payment offered must be far greater than now, or the supply will fail. It is useless to expect men of transcendent abilities to compete for the right to struggle for the earnings of a panel practitioner.

It follows that all men who practise medicine cannot be of the first class of intellect and ability, and yet the demand is that the measures to ensure good health to the community shall be under the direction of the best. An organisation must be found to be worked by the best, in which the less able may have a useful place. A skilled gardener is not necessary for every operation in the garden.

The individualistic practice of to-day is no organisation. Each man, each community, is dependent on his means, his luck, its attractions, its good or evil fortune for the man who will attend to the health of the individual, to the sanitation of the community. A wealthy resident in a rich town may be certain of having at hand a first-class man to attend him. That he often prefers a first-class humbug is partly his own fault, partly the fault of the profession. In London and in some large towns the working man can always obtain the best treatment in the world, free of all charge to himself, by subscriptions of the charitable, if he be so ill as to require treatment in a hospital. But in his home he and his family can seldom procure the best, and, since panel practice came to debase the medical profession, it often does not provide treatment as good as his meagre annual payments should procure for him. The rush necessary to make a decent living on panel terms too often leads to scamped examinations and routine empirical treatment. The less wealthy of the middle classes, and the people who dislike to receive charity in any form, notoriously find it hard to get efficient treatment, for, though the doctors they employ are able and conscientious, capable of sound advice and treatment, yet the expense of much of modern treatment is so great as to be beyond their scanty means.

Some form of State Medical Service is favoured by certain advocates, because the State already is employing great groups and small groups and individual medical men in large numbers. It is the natural course for these separated units to be organised into one whole. The State has a Navy Medical Service, an Army Medical Service, a Poor-Law Service, asylums, public health, panel, police surgeons, and so forth. Often these overlap. Often one man performs several functions. There is a deal that is chaotic. It is inevitable that some day—it seems likely it will be soon—many of these will be amalgamated into a State Medical Service. And such a Service may be a great success, will be a great success, if it does not come under the politicians and bureaucrats of a Ministry of Health and suffer the blight to all energy that comes at the breath of politics and the stagnations of bureaucracy. We are confident that the great protection of the people must be found in the persons of an army of able general practitioners, and a modern up-to-date hospital system with a paid medical staff and a pay system graded to meet the needs of all classes of people.

